# Two new species of the family Comesomatidae (Nematoda, Chromadorea, Araeolaimida) from the South China Sea

**DOI:** 10.3897/zookeys.1295.204242

**Published:** 2026-10-23

**Authors:** Shuhui Li, Lin Liu, Mian Huang

**Affiliations:** 1 College of Agriculture and Biology, Liaocheng University, Liaocheng 252059, China College of Agriculture and Biology, Liaocheng University Liaocheng China

**Keywords:** Biodiversity, *
Comesoma
*, Comesomatinae, free-living marine nematode, new species, *
Paracomesoma
*, taxonomy

## Abstract

Two new species of free-living marine nematodes found in the South China Sea are described and illustrated. *Comesoma gracilis***sp. nov**. is characterized by very slender body (a = 107.7–111); long, thick cephalic setae up to 28 µm in length; large amphidial fovea with 2.5–3 turns (86%–88% of corresponding body diameter); slender, arcuate spicules 4.5 times cloacal body diameter, with distinct transverse striations and a cephalate capitulum; plate-like gubernaculum without an apophysis; and approximately seven small papilliform precloacal supplements. This species is the only one among all known congeners with a de Man ratio *a* exceeding 107. *Paracomesoma sinica***sp. nov**. is characterized by its relatively slender body (*a* = 123–148); long, thick cephalic setae up to 25 µm in length; spiral amphidial fovea with 2.5 turns; buccal cavity cylindrical with three teeth; slender, arcuate spicules 3.4–3.5 times cloacal body diameter long, with slightly swollen proximal end; plate-like gubernaculum without apophysis; 28–30 papilliform precloacal supplements. The species can be identified from other known species within the genus by a very slender body (*a* = 123–148) and relatively shorter spicules (79–84 µm). Comparative tables of main morphometric data of valid *Comesoma* species and *Paracomesoma* species are given.

## Introduction

Free-living marine nematodes are the most dominant and diverse meiofaunal group in marine benthic habitats (Heip et al. 1982). However, a large number of nematode species are still undescribed. Nematode diversity in the marine regions of China remains largely unknown because of insufficient sampling. Currently, approximately 600 species of free-living marine nematodes have been recorded from Chinese seas, which is estimated to represent less than half of the actual species diversity (Sun et al. 2024; Sun et al. 2025b; Ma et al. 2026). Identifying nematode species is therefore very necessary.

The South China Sea, located south of the Chinese mainland, is part of the western Pacific Ocean. It is demarcated from the East China Sea by a line stretching from Nan’ao Island in Guangdong Province to the southern tip of Taiwan Island. It covers an area of approximately 3.5 million square kilometers, with an average depth of 1212 meters and a maximum depth of 5559 meters. Biodiversity surveys and taxonomic studies on nematodes in this sea region have been carried out in recent years (Lu et al. 2022; Li et al. 2024; Sun et al. 2025a). To date, because of insufficient survey sampling and a weak research foundation, approximately 500 nematode species have been identified, and a large number of species remain unknown (Sun et al. 2025a; Zhai et al. 2026). The present work describes two new species of free-living nematodes that were discovered in this sea region.

The genus *Comesoma* was established described by Bastian in 1865 with *C. vulgare* Bastian, 1865 as the type species. Currently, fifteen valid species of the genus have been accepted worldwide (Nemys 2026). However, *C. heterosetosum* Gerlach, 1955 and *C. simile* Cobb, 1898 were described only on the basis of female specimens, without male specimens. A total of five species of *Comesoma* have been reported from the sea areas of China. Among them, two species, *C. hermani* Chen & Vincx, 1998 (Wang and Guo 2023) and *C. sinica* Fu, Cai, Leduc & Lin, 2022 were recorded in the South China Sea; one species, *C. quattuordecimsupplementata* Xiao & Guo, 2023 was found in the East China Sea; two species, *C. zhangi* Sun & Huang, 2025 and *C. macramphida* Yu & Wang, 2026 were found in the Yellow Sea.

The genus *Paracomesoma* was proposed by Schuurmans Stekhoven in 1950 based on the buccal cavity with distinct teeth. *Paracomesoma coronata* Schuurmans Stekhoven, 1950 was considered a synonym of *Comesoma dubium* Filipjev, 1918, and *Comesoma dubium* was combined into *Paracomesoma dubium* (Filipjev, 1918) Schuurmans Stekhoven, 1950. Hopper (1967) reinstated the genus *Paracomesoma* Schuurmans Stekhoven, 1950, and to accommodate species of *Laimella sensu* Chitwood, 1937, viz. *Paracomesoma hexasetosa* (Chitwood, 1937), comb. nov. and *Paracomesoma quadriseta* (Chitwood, 1937), comb. nov., Hope and Murphy (1972) validated this genus name and designated *Paracomesoma coronata* Schuurmans Stekhoven, 1950 (syn. *Paracomesoma dubium* (Filipjev, 1918) Schuurmans Stekhoven, 1950) as the type species. Currently, twenty valid species have been recorded worldwide (Nemys 2026). A total of five species of *Paracomesoma* have been described in Chinese sea areas: one species, *P. heterosetosum* Zhang, 1991, found in the Bohai Sea, three species, *P. paralissum* Xiao & Guo, 2023, *P. xiamenense* Zou, 2001 and *P. zhangi* Huang & Huang, 2018 found in the East China Sea, and one species, *P. longissimum* Huang, Shi, Wang & Xu, 2021, found in the South China Sea.

## Material and methods

In October 2021, silty sediment samples from the top 0–8 cm layer were collected using a 2.9 cm diameter cut-off syringe in the intertidal zones of Huidong County along the coasts of the northern South China Sea (21°0'–22°44'N, 108°7'–115°59'E). The samples were fixed in an equal volume of 10% formalin solution. In the laboratory, samples were stained with 0.1% Rose Bengal solution for about six hours. The stained material was then passed through a set of two sieves with mesh sizes of 500 µm and 42 µm and rinsed with tap water to remove fine silt and separate macrofauna from meiofauna. The residue retained on the 42 µm mesh was centrifuged in Ludox-TM (50% colloidal silica suspension in water; Sigma Aldrich Co., USA) with a specific gravity of 1.15 g/ml (De Jonge and Bouwman 1977) to isolate meiofauna from the heavier sediment particles. The supernatant was collected after three rounds of centrifugation and filtered again through a 42 µm sieve to remove residual Ludox-TM. The retained sample was transferred into a Petri dish using distilled water, and meiofauna were sorted under a stereoscopic microscope.

Specimens were transferred to a cavity block containing a solution of 5% glycerol, 5% absolute ethanol, and 90% distilled water by volume (McIntyre and Warwick 1984). After gradual evaporation of the ethanol, the specimens were mounted in glycerin on permanent slides. The descriptions were made using a differential interference contrast microscope (Leica DM 2500), and photographs were taken with a Leica DMC 5400 digital camera. Line drawings were prepared with the aid of a camera lucida. All measurements were made using Leica LAS X software (version 3.3.3), and curved structures were measured along the midline of the curve. Type specimens were deposited at the Marine Biological Museum of the Chinese Academy of Sciences, Qingdao. Specimen records within the Material examined sections are organised first by country, then by province.

## Results and discussion

### Class Chromadorea Inglis, 1983

**Order Araeolaimida De Coninck & Schuurmans Stekhoven, 1933**

**Family Comesomatidae Filipjev, 1918**

**Subfamily Comesomatinae Filipjev, 1918**

#### Genus Comesoma

Taxon classificationAnimaliaAraeolaimidaComesomatidae

Bastian, 1865

33A12FBD-45A4-50D4-8B22-0AD2DD3D4E78

##### Diagnosis

**(emended from Fonseca and Bezerra, 2014)**. Cuticle with transverse rows of very fine punctations, without lateral differentiation. Anterior sensilla in 6+6+4 pattern; two lateral outer labial setae usually longer than the other four outer labial setae. Subcephalic setae in one or more circles, each with four setae. Amphids multispiral. Anterior portion of buccal cavity cup-shaped, posterior portion collapsed, rather weakly sclerotized and with small blunt projections at the border to anterior portion. Spicules long and slender. Gubernacular apophysis plate-like or indistinct. Precloacal supplements present or absent.

### List of valid *Comesoma* species

*C. arenae* Gerlach, 1956;

*C. bermudense* Jensen & Gerlach, 1977;

*C. hermani* Chen & Vincx, 1998;

*C. heterosetosum* Gerlach, 1955;

*C. macramphida* Yu & Wang, 2026;

*C. minimum* Chitwood, 1937;

*C. profundi* Bastian, 1865;

*C. quattuordecimsupplementata* Xiao & Guo, 2023;

*C. simile* Cobb, 1898;

*C. sinica* Fu, Cai, Leduc & Lin, 2022;

*C. solum* Pastor de Ward, 1984;

*C. stenocephalum* Filipjev, 1918;

*C. tenuispiculum* Ditlevsen, 1921;

*C. vulgare* Bastian, 1865;

*C. zhangi* Sun & Huang, 2025.

#### Comesoma gracilis sp. nov.

Taxon classificationAnimaliaAraeolaimidaComesomatidae

4B9017E6-3337-59DA-8C9A-768DB50A4A09

https://zoobank.org/9BD1CDCB-C08C-4470-A90A-C8B3FDD4D0A7

Figs 1, 2; Table 1

##### Material examined.

***Holotype*** • China, 1 ♂, holotype; Guangdong Province, Huidong County, Chishaqiao coastal beach, intertidal zone; 22°26.7'N, 114°26.7'E; 0–2 cm surface silty sediment layer; slide WHSD5‑1‑4. ***Paratype*** • China, 1 ♀, paratype; same data as for holotype; slide WHSD5‑1‑4.

**Figure 1.**
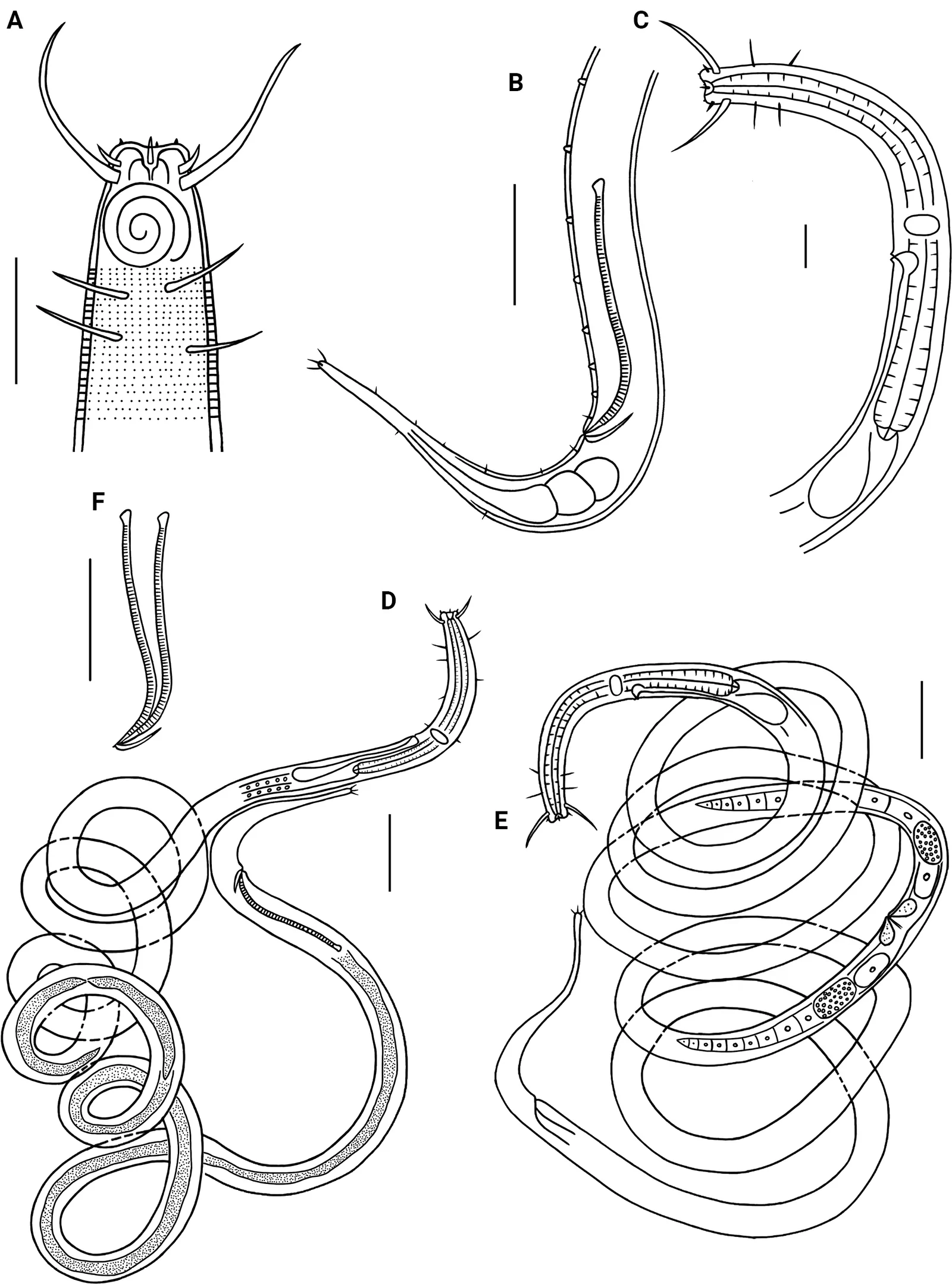
Drawings of *Comesoma gracilis* sp. nov. **A**. Lateral view of anterior part of holotype, showing labial setae, cephalic setae and amphideal fovea; **B**. Posterior end of holotype male, showing spicules, gubernaculum, precloacal supplements and caudal glands; **C**. Lateral view of anterior end of paratype, showing buccal cavity and pharynx; **D**. Entire view of holotype; **E**. Entire view of female, showing reproductive system; **F**. Spicules and gubernaculum. Scale bars: 10 µm (**C**); 20 µm (**A**); 50 µm (**B, D–F**).

**Figure 2.**
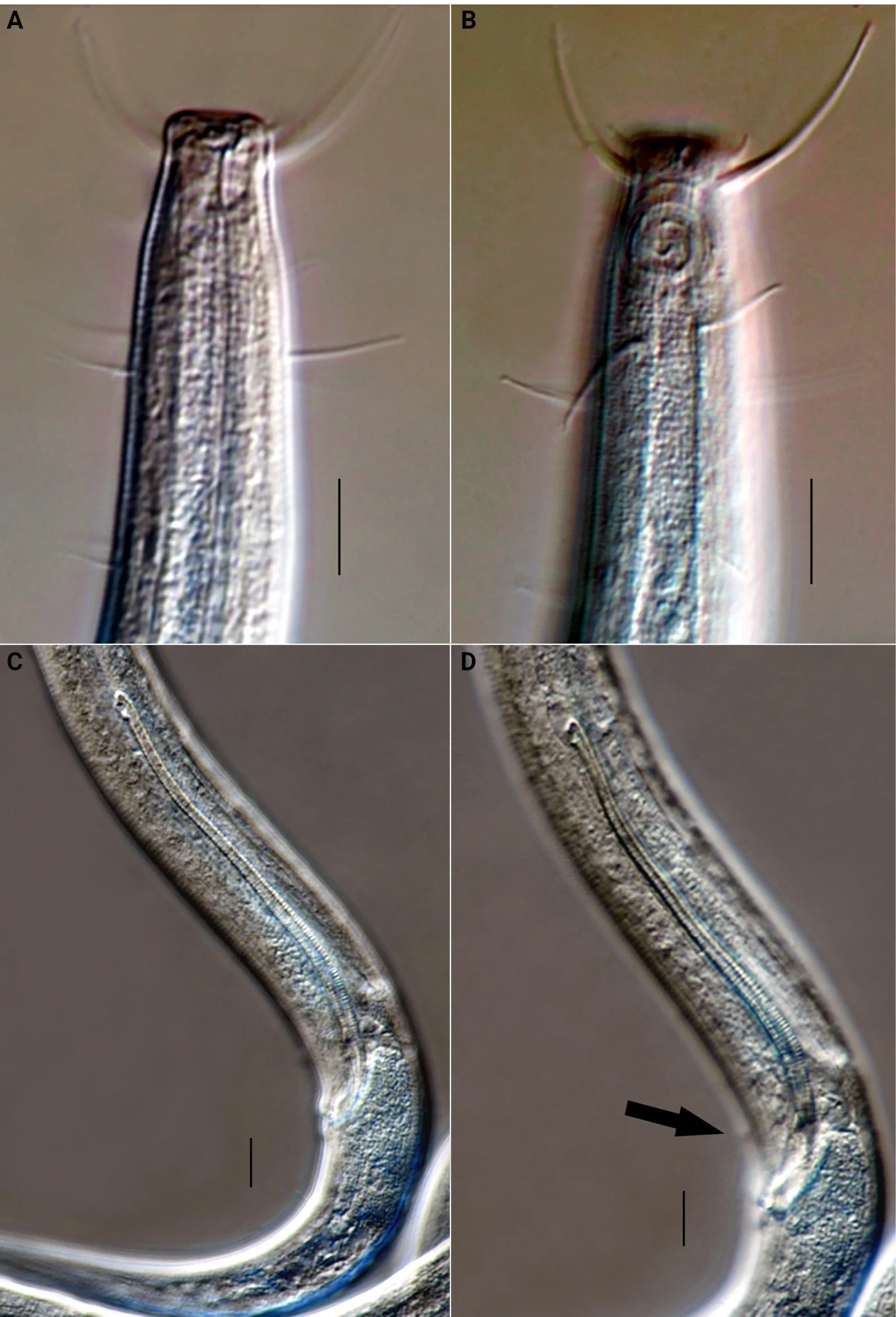
Micrographs of *Comesoma gracilis* sp. nov. **A**. Anterior end of male holotype, showing cephalic region; **B**. Anterior end of male holotype, showing outer labial setae, cephalic setae, amphideal fovea; **C**. Cloacal region of male holotype, showing spicules and gubernaculum; **D**. Cloacal region of male holotype, showing spicules, gubernaculum and precloacal supplements (arrows). Scale bars: 10 µm (**A–D**).

**Table 1.** Individual measurements of *Comesoma gracilis* sp. nov. and *Paracomesoma sinica* sp. nov. (in µm except a, b, c and V%). a: ratio of body length to maximum body diameter; At: number of turns of amphidial fovea; b: ratio of body length to pharynx length; c: ratio of body length to tail length; c': ratio of tail length to cloacal or anal body diameter; cbd: corresponding diameter of body; Cs: cephalic setae length; hd: head diameter; L: body length; Ps: number of precloacal supplements; Spic: spicules length measured as an arc; V%: position of vulva from anterior end expressed as a percentage of total body length; -: no data.

**Species**	***C. gracilis* sp. nov**.		***P. sinica* sp. nov**.			
**Morphological characteristics**	♂**1**	♀**1**	♂**1**	♂**2**	♀**1**	♀**2**
a	107.7	111.0	123.1	143.0	148.0	141.9
b	15.8	17.1	16.8	17.1	17.8	19.7
c	19.0	18.9	18.3	19.5	14.9	18.2
c'	5.9	7.7	8.1	7.3	12.8	10.0
Total body length	2585	2775	3570	3290	3995	3832
Maximum body diameter	24	25	29	23	27	27
Head diameter	11	9	11	12	12	11
Length of labial setae	4	3	5-12	5-10	5-12	4-11
Length of cephalic setae	24	28	21-25	23	25	24
Width of amphidial fovea	11.5	9.5	8	-	8	8
Body diameter at amphid	13	11	10.5	-	13	12
Excretory pore from anterior end	101	95	146	126	-	136
Nerve ring from anterior end	86	98	130	112		110
Length of pharynx	164	162	213	192	224	195
Body diameter at pharyngeal base	21	21	24	22	24	22
Spicules length along arc	103	-	84	79	-	-
Length of gubernaculum	18	-	15	15	-	-
Number of precloacal supplements	7	-	30	28	-	-
Tail length	136	147	195	169	268	210
Body diameter at cloaca or anus	23	19	24	23	21	21
Vulva from anterior end	-	1424	-	-	2137	1928
cbd of vulva	-	25	-	-	27	27
V%	-	51	-	-	53	50

##### Etymology.

The species epithet is derived from a Latin adjective referring to its relatively slender body.

##### Measurements.

All measurement data are given in Table 1.

##### Description.

**Male**. Body slender, gradually narrowing toward both ends. Cuticle with transverse rows of fine punctation starting from posterior half of amphid to near tail tip. Lateral differentiation absent. Head blunt, slightly set off by a contraction at the level of cephalic setae. Head diameter (body width at the level of cephalic setae) 11 µm. Anterior sensilla in three separate circles; six inner labial sensilla papilliform, six outer labial sensilla setiform, 4 µm long; and four cephalic setae very long and thick, 24 µm long, i.e., 2.2 times head diameter, following closely behind outer labial setae. Two circles of subcephalic setae (12–13 µm long) situated below the amphids, twice the head diameter from the anterior end, each circle with four setae. Somatic setae short and scattered, present in pharyngeal and caudal regions. Amphidial fovea multispiral with 3 turns, 11.5 µm wide, and occupying 88% of corresponding body diameter, located immediately posterior to the circle of cephalic setae. Buccal cavity small, cup-shaped, without tooth with posterior portion narrowed. Pharynx cylindrical, gradually enlarging posteriorly but not forming true posterior bulb. Nerve ring located at about mid-length of the pharynx. Cardia conical, 7 µm long. Secretory-excretory system consisting of a large unicellular gland cell located 50 µm posterior to the pharyngeal base; secretory-excretory duct with distinct ampulla and opening posterior to the nerve ring, i.e., 101 µm from anterior end. Tail conico-cylindrical, the anterior two-thirds conical, and the posterior one-third cylindrical. Tail tip slightly swollen, with three terminal setae, 7 µm long. Other caudal setae 3 µm long. Three caudal glands and spinneret present.

Reproductive system diorchic, with two opposed, outstretched testes. Anterior testis to the left of the intestine and posterior testis to the right. Spicules slender and arcuated, approximately 4.5 times as long as cloacal body diameter in length, with a cephalate capitulum and a pointed distal end, and evenly distributed transverse striations from the distal end to the proximal end. Gubernaculum simple, plate-like, 18 µm long, without apophysis. Seven small papilliform precloacal supplements. Spacing between adjacent supplements gradually increasing anteriorly. The most anterior supplement located 136 µm from the cloaca and the posterior one 13 µm anterior to the cloaca. One short precloacal seta located immediately anterior to the cloaca (5 µm from cloaca), 3 µm long.

**Female**. It is similar to male in general characteristics, but the cephalic setae longer (28 µm long), amphidial fovea smaller (2.5 turns, 9.5 µm wide), tail slightly longer (7.7 anal body diameter). Reproductive system having two opposed, outstretched ovaries. Anterior ovary to the left of the intestine and posterior one to the right. Vagina short with thick walls. Two spermathecae present, oval sac-shaped structures, situated anterior and posterior to the vulva, respectively. The anterior spermatheca lies 2 vulval body diameters from the vulva, and the posterior one approximately 1.8 vulval body diameters from the vulva. Vulva outwardly protruding, located at mid-body.

##### Diagnosis and discussion.

*Comesoma gracilis* sp. nov. is characterized by very slender body (a = 107.7–111); long, thick cephalic setae (up to 28 µm in length); large amphidial fovea with 2.5–3 turns (86%–88% of corresponding body diameter); slender, arcuate spicules approximately 4.5 times the cloacal body diameter, with distinct transverse striations and a cephalate capitulum; plate-like gubernaculum without apophysis; seven small papilliform precloacal supplements.

*Comesoma gracilis* sp. nov. is similar to *C. zhangi* in body size, long and thick cephalic setae, and spicules length, but differs from the latter species by extremely slender body (a = 107.7–111 vs 37–46.3), outer labial sensilla setiform (vs papilliform), 2 rows of 4 subcephalic setae posterior to amphids (vs anterior to amphids), amphidial fovea with three turns in males, 2.5 turns in female (vs 2 turns in both male and female), and spicules having distinct transverse striations and cephalate proximal end. The new species is also similar to *C. macramphida* Yu & Wang, 2026 in body size and possession of long cephalic setae, but differs by shorter outer labial setae (3–4 µm vs 6 µm), amphidial fovea with 3 turns in male (vs 2.5 turns in males), shorter pharynx (de Man ration *b* = 15.8–17.1 vs 12.1–12.9), longer spicules (4.5 vs 2.21–2.36 cloacal body diameters), and fewer precloacal supplements (7 vs 10–11). *Comesoma gracilis* sp. nov. is distinguished from other congeners by its extremely slender body, amphidial fovea with three turns in males, and spicules with distinct transverse striations. The difference between *Comesoma gracilis* sp. nov. and other described species in the genus can be inferred from Table 2.

**Table 2.** Comparative table of main morphometric data of valid *Comesoma* species and the new species (according to Xiao and Guo 2023) and subsequent literature. L: body length; Cs: Cephalic setae length; At: number of turns of the amphidial fovea; Spic: spicule length as an arc; Nps: number of precloacal supplements; -: no data (µm in length).

**Species**	**L**	**Cs**	**At**	***a***	***b***	***c***	***c'***	**Spic**	**Nps**
*C. arenae*	2180	23	2.0	36	7.8	12.1	3.9	273	-
*C. bermudense*	2380–3035	27–38	2–2.5	32–46	8.2–11.4	8.5–11.3	4.1–5.4	150–194	18–22
*C. hermani*	1694–2108	13	2.5	30.3–40.9	7.6–9.1	9.5–12.1	4–4.5	169–184	present
*C. heterosetosum*	2219–3480	42	2.0	57–73	10.1–14.8	13.4–19.8	4.8–5.3	-	-
*C. macramphida*	2753–2933	23–30	2.5	78.7–104.8	12.1–12.9	16.8–18.8	5.4–6.6	59–64	10–11
*C. minimum*	1370–1520	1.1 hd	2.3	23–24	7.7	8.2	8.2–9	6.3 abd	present
*C. profundi*	2800	-	-	37.0	11.1	-	3.9	178	-
*C. quattuordecimsupplementata*	1293–1498	9–10	2.5–2.75	23.3–30.6	7.3	9–9.7	4.2–4.4	120–128	14
*C. simile*	3300	26	-	-	-	-	-	-	-
*C. sinica*	2366–2925	24–32	2.5	34–52	10–12	12–17	3–5	166–179	20
*C. solum*	3400	25	1.75	26.1	11.3	13.6	3.1	270	9
*C. stenocephalum*	4600	22	2.3	55	14	14	5.3	165	-
*C. tenuispiculum*	1600	1.5 hd	2.5	42	8.7	9.0	3.5	112	-
*C. vulgare*	3600	-	-	31.7	9.1	-	5.0	198	-
*C. zhangi*	2182–2500	27–28	2	37–46.3	10.7–11.6	11.5–12.8	5–5.4	101	absent
*C. gracilis* sp. nov.	2585–2775	24–28	2.5–3	108–111	15.8–17.1	18.9–19.0	5.9–7.7	103	7

Regrettably, only one male and one female were found in the samples, suggesting that this is a rare species. Although the number of specimens is limited, the morphological characteristics of the present species are distinct and allow clear distinction from its congeners. The morphological data of all known valid *Comesoma* species, together with those of the new species, are presented in Table 2.

#### Genus Paracomesoma

Taxon classificationAnimaliaAraeolaimidaComesomatidae

Schuurmans Stekhoven, 1950

85AB31F5-DB28-5615-9BC0-7573756A3E15

Paracomesoma . – Hope & Murphy, 1972.

##### Diagnosis

**(from Huang et al. 2021)**. Multispiral amphideal fovea with 2–3 turns. Anterior sensilla in three distinctly separated circles; six outer labial sensilla may be unequal in length. Cuticle with transverse striations or punctations. Lateral differentiation present or absent. Posterior portion of the buccal cavity cylindrical, with sclerotized walls and provided with three or six thorn-like projections at the border of the anterior portion. Spicules long and slender. Gubernaculum plate-like without dorsal apophysis. Precloacal supplements usually present.

### List of valid *Paracomesoma* species

*P. curvatum* Gagarin & Thanh, 2006;

*P. curvispiculum* (Allgén, 1959) Jensen, 1979;

*P. dubium* (Filipjev, 1918) Schuurmans Stekhoven 1950;

*P. elegans* Gagarin & Thanh, 2009;

*P. heterosetosum* Zhang, 1991;

*P. hexasetosum* (Chitwood, 1937) Hopper 1967;

*P. inaequale* Jensen & Gerlach, 1977;

*P. leptum* Gagarin & Tu, 2018;

*P. lissum* Gagarin & Thanh, 2009;

*P. longispiculum* (Timm, 1961) Hopper 1967;

*P. longissimum* Huang, Shi, Wang & Xu, 2021;

*P. minor* Gagarin & Tu, 2014;

*P. paralissum* Xiao & Guo, 2023;

*P. paralongispiculum* Tu, Vanreusel, Smol, Long & Thanh, 2013;

*P. quadrisetosum* (Chitwood, 1937) Hopper 1967;

*P. sigmoidalis* Riera, Nunez & Del Carmen Brito, 2006;

*P. sipho* (Gerlach, 1956) Jensen and Gerlach 1977;

*P. susannae* Semprucci, 2014;

*P. xiamenense* Zou, 2001;

*P. zhangi* Huang & Huang, 2018.

#### Paracomesoma sinica sp. nov.

Taxon classificationAnimaliaAraeolaimidaComesomatidae

AF0B122E-B57E-5CB0-AF79-DB1F4756CE4A

https://zoobank.org/5346EB66-1A22-40ED-90EE-919F7258EFE9

Figs 3, 4; Table 1

##### Material examined.

***Holotype*** • China, 1 ♂, holotype; Guangdong Province, Huidong County, Chishaqiao coastal beach, intertidal zone; 22°26.7'N, 114°26.7'E; 0–2 cm surface silty sediments; slide WHSD5‑1‑4. ***Paratypes*** • China, 1 ♀, paratype; same data as for holotype; slide WHSD5‑1‑4. • China, 1 ♂, paratype. • CHINA, 1 ♀, paratype.

**Figure 3.**
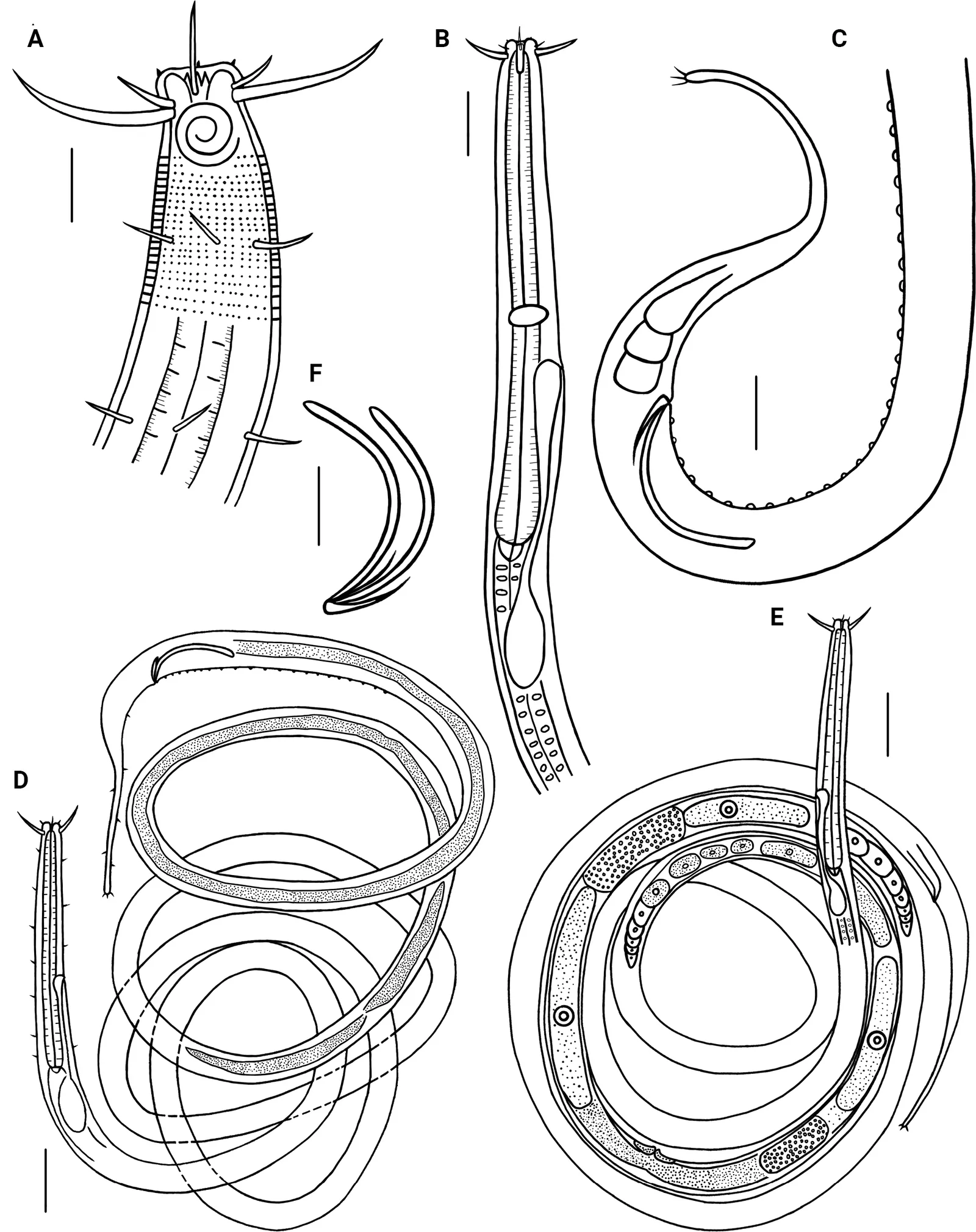
Drawings of *Paracomesoma sinica* sp. nov. **A**. Lateral view of anterior part of holotype, showing labial setae, cephalic setae, amphideal fovea and pharynx; **B**. Lateral view of anterior end of paratype, showing buccal cavity and pharynx; **C**. Posterior end of holotype male, showing spicules, gubernaculum, precloacal supplements and caudal glands; **D**. Entire view of holotype; **E**. Entire view of female, showing reproduction system; **F**. Spicules and gubernaculum. Scale bars: 10 µm (**A, C**); 20 µm (**B**); 30 µm (**D, E**); 50 µm (**F**).

**Figure 4.**
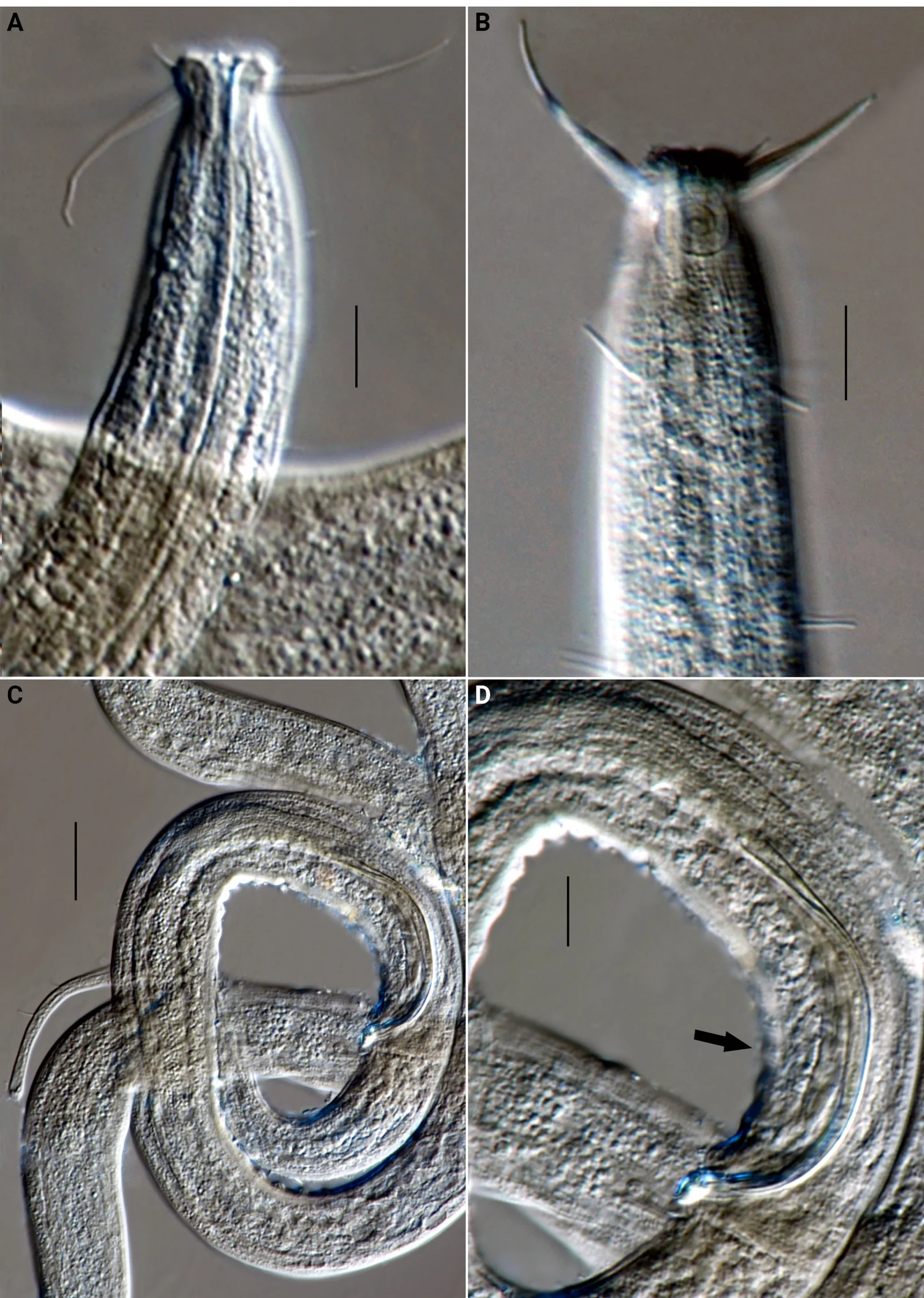
Micrographs of *Paracomesoma sinica* sp. nov. **A**. Anterior end of male holotype, showing cephalic region; **B**. Anterior end of male holotype, showing outer labial setae, cephalic setae, amphideal fovea; **C**. Cloacal region of male holotype, showing spicules and gubernaculum; **D**. Cloacal region of male holotype, showing spicules, gubernaculum and precloacal supplements (arrows). Scale bars: 10 µm (**A, B, D**); 20 µm (**C**).

##### Etymology.

The species epithet *sinica* is derived from the Latin adjective referring to the country of origin, China.

##### Measurements.

All measurement data are given in Table 1.

##### Description.

**Males**. Body slender, Cuticle with transverse rows of fine punctations, without lateral differentiation. Head set off by a contraction at the level of cephalic setae. Head diameter 11–12 μm. Head sensilla arranged in 6+6+4 pattern. Inner labial sensilla papilliform; six outer labial sensilla setiform, unequal in length, with the two lateral ones long (10–12 µm long) and the remaining four short (5 µm long). Four cephalic setae very long and thick, 21–25 µm long, i.e., 1.9–2.3 times head diameter. Cervical setae 8–10 µm long, arranged in six longitudinal rows, six setae per circle. Somatic setae only present in pharyngeal and caudal regions. Amphidial fovea spiral with 2.5 turns, 8 µm wide, occupying about 76% of corresponding body diameter, located immediately posterior to the circle of cephalic setae. Buccal cavity cylindrical, 13 µm deep and 2 µm wide, with one dorsal and two subventral small teeth. Pharynx cylindrical, gradually enlarging posteriorly but not forming true posterior bulb. Cardia conical, 13 µm long. Nerve ring situated post-mid pharynx, 58%–61% of the pharynx length from the anterior end. Secretory-excretory system consisting of a large unicellular gland cell located 60 µm posterior to the pharyngeal base; secretory-excretory duct with a distinct ampulla and opening below the nerve ring, i.e., 126–146 µm from the anterior end. Tail slender, conico-cylindrical, 7.3–8.1 cloacal body diameter long, conical in the anterior half and cylindrical in the posterior half. Tail tip slightly swollen, with three terminal setae, 6 µm long. Caudal setae 5–6 µm long. Three caudal glands and spinneret present.

Reproductive system having two opposed and outstretched testes. Anterior testis to the left and posterior testis to the right of the intestine. Spicules paired, equal and arcuated, 3.4–3.5 times as long as cloacal body diameter, with slightly swollen proximal end, without distinct capitulum. Gubernaculum plate-like, 15 µm long, without apophysis. Precloacal supplements consist of 28–30 nearly equidistant papilliform structures. The most anterior supplement located 180–190 µm from the cloaca, and the posterior one 10 µm anterior to the cloaca.

**Females**. Similar to males in general characteristics, except body and tail slightly longer (10–12.8 anal body diameter). Reproductive system having two opposed, outstretched ovaries. Anterior ovary to the left of the intestine and posterior one to the right. Vagina short with thick walls. Two spermathecae situated anterior and posterior to the vulva, respectively. Anterior spermatheca lies 3 vulval body diameters from vulva, and posterior one 5–6 vulval body diameters from vulva. Eggs large, elongate- oblong, up to 250 µm in length and 18 µm in width. Vulva outwardly protruding, located at mid-body, i.e., 50%–53% body length from the anterior end.

##### Diagnosis and discussion.

*Paracomesoma sinica* sp. nov. is characterized by its relatively slender body (a = 123–148); long, thick cephalic setae (up to 25 µm in length); spiral amphidial fovea with 2.5 turns; buccal cavity cylindrical with three teeth; spicules slender, arcuate, relatively short within the genus, 3.4–3.5 times the cloacal body diameter long, with slightly swollen proximal end; plate-like gubernaculum without apophysis; presence of 28–30 papilliform precloacal supplements.

*Paracomesoma sinica* sp. nov. is similar to *P. heterosetosum*, *P. inaequale* and *P. longissimum* in body size and shape. However, the new species differs from *P. heterosetosum* by the fewer turns of the amphidial fovea (2.5 vs 3.5 turns) and shorter spicules (79–84 µm vs 188–205 µm). The new species differs from *P. inaequale* in having shorter cephalic setae (21–25 µm vs 36–46 µm) and shorter spicules (79–84 µm vs 148–175 µm); differs from *P. longissimum* by shorter spicules (79–84 µm vs 311–360 µm), and a greater de Man ratio *a* value (123–148 vs 94.8–96.7). In having relatively short spicules (shorter than 100 µm), the new species is similar to *P. hexasetosum*, *P. lissum*, *P. minor* and *P. quadrisetosum*, but it can be distinguished from the latter four species by its longer body length (3290–3995 µm vs shorter than 2300 µm of the latter four species), very large de Man *a* value (123–148 vs less than or equal to 50), and relatively longer tail (c'=7.3–12.8 vs equal or less than 6). The differences between *Paracomesoma sinica* sp. nov. and other known species within the genus are shown in Table 3.

**Table 3.** Comparative table of main morphometric data of valid *Paracomesoma* species and the new species according to Semprucci (2014) and Xiao and Guo (2023 and other relevant records. L: body length; Cs: Cephalic setae length; At: The number of turns of the amphidial fovea; Spic: Spicules length as arc; Nps: Number of precloacal supplements; -: no data (µm in length).

**Species**	**L**	**Cs**	**At**	***a***	***b***	***c***	***c'***	**Spic (µm)**	**Spic/cbd**	**Nps**
*P. curvatum*	1750–1970	5.0–5.5	3	28–40	8.8–10.0	9.9–12.4	4.1–5.1	300–343	7.5-9.7	18–25
*P. curvispiculum*	2127	-	2	42.5	10.4	16.8	-	3.9 abd	3.9	-
*P. dubium*	2800	17	2	49.0	11.5	14.0	-	175	2.9-3.9	40
*P. elegans*	1042–1218	3.5–4.5	3	27–46	8.0–9.9	10.5–13.2	3.5–5.1	125–165	4.6	8–12
*P. heterosetosum*	2754–3860	8–36	3.5	89–121	11–16	16–22	5.9–7.1	188–205	6.4-7.5	26–32
*P. hexasetosum*	2220	1.5 hd	2.75	50.0	10.0	13.3	-	2 abd	2.0	≥ 40
*P. inaequale*	4290–5190	36–46	2.5–3	103–124	14.6–17.2	14.7–18	-	148–175	3.8-4.6	25–32
*P. leptum*	1025–1227	9–13	2.5–2.8	26–35	6.0–7.2	8.3–11.1	3.5–4.5	102–123	3.7	9–13
*P. lissum*	1338–1495	6.5–7	3	33–35	7.8–8.9	9.8–10.4	4.2–4.7	88–98	3.2	45–47
*P. longispiculum*	1500	8	2.5	28.0	7.6	9.4	-	225	6.0	≥ 50
*P. longissimum*	2514–2750	20–32	2.5	94.8–96.7	12.2–13.4	16.4–17.8	6.1–7.3	311–360	15.7	25–28
*P. minor*	961–977	14–14.5	3.5	30.0–38.0	7.2–7.4	8.6–8.7	5.0–5.7	93–98	3.2	8–9
*P. paralissum*	1815–1841	4.3–5.8	3	26.7–29.2	8.1–8.8	7.7–7.8	5.1–5.3	126	2.7-2.8	40
*P. paralongispiculum*	1785–1989	4.8–6.3	2.5	41.6–47.1	9.8–11.3	11.2–13.3	5.1–5.5	344–361	9.2-10.5	11–12
*P. quadrisetosum*	1440–1480	1.2 hd	2.3	31.0–40.0	8.2–8.9	9.0–11.0	-	2 abd	2.0	≥ 30
*P. sigmoidalis*	2343–2657	30–39.3	3.5	37–53.1	7.7–10.8	7.9–15	2.4–4.8	164–271	3.6-5.2	35
*P. sipho*	2310	33	2.5	51.0	8.5	10.0	-	160	5.0	30
*P. susannae*	3542–4455	72–91	2.5	59.0–71.7	14.5–18.7	13.0–17.3	6.7	117–120	2.3-2.9	19–22
*P. xiamenense*	1700–2000	8.9	-	37.9–45.3	6.7–8.3	7.6–10.1	6.0–7.0	188–203	5.8	28–30
*P. zhangi*	1868	13.5	3	42.5	8.7	9.9	5.4–6.1	152	4.3	26
*P. sinica* sp. nov.	3290–3995	21–25	2.5	123–148	16.8–19.7	14.9–19.5	7.3–12.8	79–84	3.4-3.5	28–30

## Supplementary Material

XML Treatment for Comesoma

XML Treatment for Comesoma gracilis

XML Treatment for Paracomesoma

XML Treatment for Paracomesoma sinica

## Additional information

### Artificial Intelligence (AI) use

The authors accept full responsibility for the content of the manuscript, including the disclosure of any use of AI.

No AI tools were used in the preparation of this manuscript.

### Author ORCIDs

S. Li https://orcid.org/0009-0008-2520-2297

L. Liu https://orcid.org/0009-0005-7503-6942

M. Huang https://orcid.org/0000-0003-3343-1520

### Data availability

All of the data that support the findings of this study are available in the main text.
